# Specialists in Medicine

**Published:** 1863-05

**Authors:** 


SPECIALISTS IN MEDICINE.—The Commissioners of Charities and Corrections of this city have addressed a communication to the Medical Board of Bellevue Hospital inquiring as to the propriety of establishing specialities in that hospital, instancing diseases of the eye, the ear, the nervous system and skin diseases. They inquire also as to the propriety of introducing other specialties. The inference would seem to be that the diseases in the hospital might be classified according to the modern views of specialists, by arranging a large number of sub-divisions.

The questions proposed to the Medical Board by the Commissioners being prominently before the profession the much agitated subject of specialties in practical medicine. We trust it will receive the dispassionate consideration of that body, for on its decision will depend, to a certain extent, the initiative movements of specialism in this country It should also receive, in a wider and more general sense, the earnest attention of the profession at large, and such policy should be adopted for the encouragement or discouragement of these special studies as will best promote the interests of medicine.

The tendency of the age is undoubtedly to the division of labor. We see it in all the mechanic arts and in every department of human labor and thought. It grows out of the limited powers of the human body and mind, and the constant expansion of every branch of science and of every department of industry. Not all minds can grasp the widely varying facts in any one of the generally recognized divisions of the sciences, much less become profoundly acquainted with them. Neither can the artizan become proficient in many branches of the same business. He who is recognized as a "Jack at all trades" can not excel in any one art, though he may be a most useful person by his general knowledge.

Medicine, as a science and an art, has not escaped this tendency to the division of labor. The three grand divisions, practical medicine, surgery, and obstetrics, have long been recognized and adopted. They very naturally grow out of fundamental differences in methods of treatment of diseases. Surgical affections require, for the most part, entirely different remedial agents from medical disease; and the practice of obstetrics differs equally from both in the appliances of art. In the progress of the medical sciences many of the classes of diseases embraced in these several grand divisions have become objects of special study, as the diseases of the eye and the ear in surgery, of the heart and lungs in medicine, and of the uterus in obstetrics. These divisions have gradually become more and more numerous, until surgery, medicine and obstetrics are little else than an agglomeration of specialties. The necessity of pursuing the study and subsequent practice of a specialty in order to success, is beginning to possess the minds of young medical men. They are stimulated by the examples of men who have won reputation and fortune by devotion to a specialty. It is time that this subject was thoroughly discussed in all its bearings, that the younger members of the profession may have correct views of the advantages or disadvantages of pursuing a specialty.

The arguments generally brought forward by the advocates of specialties in medicine are, as we have already intimated, those which apply to a division of labor in any other department of business. And they have great plausibility. To surpass all contemporary laborers in any special pursuit requires the undivided efforts of every ordinary mind. But we are not prepared to accept this reasoning in an unqualified sense. As a general fact we must aver that the man whose knowledge in business takes the widest range has the best basis for success. This fact is eminently true in medicine. The general surgeon, physician, or obstetrician, will prove a better practitioner in any particular disease than the specialist. The most successful surgeon is the man who has also a thorough knowledge of practical medicine. The relations of diseases are intimate, often obscure, and frequently all-controlling. The practitioner who fails to detect these relations will certainly not be a good practitioner, whatever may be his pecuniary success. He will treat diseases in his specialty from the most narrow stand point, and too frequently fail to comprehend those remote agencies and widely extended sympathies which most seriously modify their progress. These peculiarities the general practitioner anticipates and readily recognizes, and promptly meets every manifestation with proper remedies.

It has been remarked by an accurate observer of the progressive changes in the medical profession, that the older practitioners often much excel the younger in the treatment of disease because they take a much wider range of symptoms, and do not narrow their view to single organs and individual diseases. There is much truth in the remark. The recent graduate has his mind pre-occupied with scholastic divisions and subdivisions of diseases of individual parts, and in his analysis he becomes more and more restricted, until the attention is fixed upon a limited, perhaps an insignificant, part of the subject under investigation. The same is true of the pure specialist, as the term is now employed. In general he is necessarily a poor practitioner, and though he may more correctly interpret the signs of pathological changes than others who have studied the special forms of disease less minutely, he will show but little skill in employing remedies. If we contrast also the usefulness and the rank in the profession of the general practitioner with that of the specialist, we shall see clearly that the studies of the former tend much more to enlarge the mind, to strengthen its grasp, and increase its powers of correct analysis.

We shall not pursue the subject further at this time. We are glad that it has been presented to the Medical Board of one of the largest hospitals in the country, and we await its decision with great interest. If any hospital is susceptible of easy classification of the diseases of the inmates, Bellvue may be taken as better adapted than any other; and it is proper that the experiment should be first attempted within its walls.—*American Medical Times.*

